# Prevalence of new variants of *Chlamydia trachomatis* escaping detection by the Aptima Combo 2 assay, England, June to August 2019

**DOI:** 10.2807/1560-7917.ES.2019.24.38.1900557

**Published:** 2019-09-19

**Authors:** David J Roberts, Grahame S Davis, Michelle J Cole, Dixita Naik, Hitiksha Maru, Neil Woodford, Peter Muir, Paddy Horner, Ian Simms, George Thickett, Paul Crook, Kirsty Foster, Nick Andrews, John Saunders, Helen Fifer, Kate Folkard, O Noel Gill, on behalf of the incident management team

**Affiliations:** 1 National Infection Service, Public Health England, London, United Kingdom; 2 South West Regional Public Health Laboratory, Public Health England, Bristol, United Kingdom; 3 Population Health Sciences, University of Bristol, Bristol, United Kingdom; 4 Field Service South East and London, Public Health England, London, United Kingdom; 5 Public Health England North East Centre, Newcastle-upon-Tyne, United Kingdom; 6 Statistics, Modelling and Economics Department, Public Health England, London, United Kingdom; 7 Members are listed in the acknowledgements

**Keywords:** Chlamydia trachomatis, nucleic acid amplification testing, epidemiology

## Abstract

We identified two new *Chlamydia trachomatis* (CT) variants escaping Aptima Combo 2 (AC2) assay detection, in clinical specimens of two patients. One had a C1514T mutation the other a G1523A mutation, both within the AC2 23S rRNA target region. The prevalence of such variants among persons tested for CT in England was estimated to be fewer than 0.003%.

At the end of April 2019, Public Health England (PHE) was alerted, via an international Epidemic Intelligence System-Sexually Transmitted Infections (EPIS-STI) post from Finland, of false-negative *Chlamydia trachomatis* (CT) test results using the Aptima Combo 2 (AC2) assay (Hologic Inc., San Diego, California, United States (US)), a nucleic acid amplification test (NAAT) for CT (target: 23S rRNA) and *Neisseria gonorrhoeae* (GC) (target: 16S rRNA). Discrepant results between the AC2 assay and the Aptima *Chlamydia trachomatis* assay (ACT) (target: 16S rRNA) were reported to have occurred primarily in specimens that had AC2 relative light units (RLU) from 20 to 84 [1]. These false-negative AC2 results [2,3] were attributed to a C1515T mutation in the CT 23S rRNA gene. In early June 2019, Hologic Inc. issued a Field Safety Notice (FSN) to AC2-using laboratories, recommending ACT reflex retesting of AC2 CT-negative with RLU ≥ 15, CT-equivocal, or GC-equivocal/-positive (if CT-negative/equivocal) specimens to ensure detection of the Finnish new variant CT strain (F-nvCT) [3]. A European Centre for Disease Prevention and Control (ECDC) rapid risk assessment recommended countries estimate the spread of F-nvCT to inform the need for patient recall and retesting [4]. Here we report results from an investigation coordinated by a multiagency incident management team (IMT) to ascertain the prevalence of new variants of *Chlamydia trachomatis* escaping detection by the Aptima Combo 2 assay in England.

## Initial response and risk assessment

The IMT was informed by Hologic Inc. that 42 laboratories in the nine PHE regions in England, one in Isle of Man (IoM) and one in Scotland were using the AC2 assay. De-duplicated test data from laboratories in England extracted from the CTAD *Chlamydia* surveillance system [5] showed AC2 assays accounted for 44% (1,684,028/3,863,336) of CT tests nationally in 2018.

In undertaking the risk assessment, a local prevalence threshold of 0.1% was chosen by the IMT as a balance between confidence that any health risk would be minimal and the amount of data that could be obtained from all regions within a short timeframe. To inform whether a patient recall was indicated, the IMT estimated the F-nvCT prevalence in each region by: (i) asking laboratories in England/IoM (IoM was included within the North West region) using AC2 to forward weekly AC2 testing and retesting data to the IMT (specimens eligible for retest were defined as per the FSN, weekly AC2 tests reported were compared with April 2018 to March 2019 average weekly AC2 tests to estimate the likely completeness of laboratory reporting); (ii) asking laboratories using AC2 to refer discrepant specimens (retested specimens found to have a positive result using ACT) for reference laboratory investigation; (iii) undertaking partial 23S rRNA gene Sanger sequencing [1] to detect mutations in the AC2-CT target region; and (iv) calculating the one-sided exact-binomial upper 95% confidence interval for new variant CT AC2-negative (nvCT/AC2neg) prevalence among those tested.

## Prospective *Chlamydia trachomatis* testing and retesting data

Four of 43 laboratories expected to report did so via the same central laboratory, five laboratories confirmed they were not using the test, and two laboratories not originally indicated by Hologic Inc. to be using the AC2 test also reported data or sent discrepant specimens. By 18 August 2019, 31 of 37 laboratories using AC2 reported on 242,505 tests from 1 June 2019. This was 79% of the estimated expected number of tests (n = 307,779) based on historical laboratory throughput (Table 1). From 1 June to 18 August 2019, 14,075 of 242,505 (5.8%) specimens were CT-positive on the AC2 test and 11,518 of 242,505 (4.7%) were retested (Table 2). PHE’s reference laboratory received 266 discrepant specimens from 26 laboratories.

**Table 1:** Aptima Combo 2 (AC2) *Chlamydia trachomatis* tests expected and reported by region, England, 1 June^a^–18 August 2019 (n = 37)

Region	Laboratories using AC2	Laboratories using AC2 and reporting in period	Weekly AC2 tests expected	Number of reporting weeks	Estimated AC2 tests expected in period^b^	AC2 tests reported	AC2 tests reported as percentage of estimated (%)
East Midlands	2	2	990	10	9,757	7,355	75
East of England	5	3	1,165	10	11,650	3,077	26
London	5	4	13,680	9	123,117	82,753	67
North East	1	1	563	7	3,862	3,966	103
North West^c^	7	7	4,221	11	47,030	65,935	140
South East	5	3	3,438	8	27,504	10,471	38
South West	4	4	3,353	10	33,054	31,510	95
West Midlands	3	3	3,245	7	23,641	23,747	100
Yorkshire and Humber	5	4	3,180	9	28,164	13,691	49
**Total**	**37**	**31**	**33,834**	**NA**	**307,779^d^**	**242,505**	**79**

NA: not applicable.

^a ^Two Public Health England laboratories were asked to initiate retesting before the Hologic Inc. Field Safety Notice [3].

^b^ The values in the column may not match the product of weekly AC2 tests expected multiplied by reporting weeks because of the rounding of AC2 expected weekly tests and reporting weeks.

^c^ Isle of Man included in North West subtotals.

^d^ The total for all of England is calculated from the sum of estimated AC2 tests expected in the period, not the product of weekly AC2 tests expected multiplied by reporting weeks.

**Table 2:** Results of retesting Aptima Combo 2 (AC2) *Chlamydia trachomatis* specimens with Aptima *Chlamydia trachomatis* assay (ACT)^a^ by region, England, 1 June^b^–18 August 2019

Region	AC2 tests reported	AC2 tests retested by ACT	Re-test percent (%)	Discrepants received^c^
East Midlands	7,355	78	1.1	4
East of England	3,077	39	1.3	0
London	82,753	6,004	7.3	74
North East	3,966	12	0.3	3
North West^d^	65,935	3,774	5.7	62
South East	10,471	307	2.9	30
South West	31,510	392	1.2	32
West Midlands	23,747	688	2.9	29
Yorkshire and Humber	13,691	224	1.6	32
**Total**	**242,505**	**11,518**	**4.7**	**266**

^a^ Specimens eligible for retest were defined as per the Hologic Inc. Field Safety Notice [3].

^b ^Two Public Health England laboratories were asked to initiate retesting before the Hologic Inc. Field Safety Notice [3].

^c^ Refers to discrepant specimens received by Public Health England Colindale for reference laboratory investigations.

^d^ Isle of Man included in North West subtotals.

Noticeably higher retesting rates were seen in London and the North West (Table 2). This was considered due to factors such as operational differences between laboratories and higher rates of gonorrhoea diagnosis [6]. In accordance with the FSN, samples that were positive for gonorrhoea were retested.

## Reference laboratory analyses

By 18 August 2019, reference laboratory investigations had been completed on 209 of 266 (79%) of the discrepant specimens received by PHE. The first 19 specimens were referred to the World Health Organization Collaborating Centre for Gonorrhoea and Other STIs at Örebro University, Sweden and the remainder processed within PHE. The C1515T mutation was not detected. However, two new mutations were detected. The first was a C1514T mutation that was detected in July from one symptomatic male (two specimens) in their 20s with prior heterosexual contacts in the US. The second was a G1523A mutation that was also detected in July in a specimen from an asymptomatic female in their 20s; no information on sexual contacts was available. Neither mutation was recorded in the US National Center for Biotechnology Information’s GenBank. These two nvCT/AC2neg cases originated from 242,505 AC2 tests reported by laboratories using AC2 between 1 June and 18 August 2019. The sequences derived from these two cases will be deposited into GenBank.

Assuming the AC2 test data and referred specimens were broadly representative of the population, and that very few individuals would have had duplicate specimens in the period, the upper 95% confidence interval boundary for nvCT/AC2neg prevalence among those tested in England was calculated to be 0.003% (1 in 38,519), ranging from 0.004% to 0.097% by region, and 0.001% nationally for F-nvCT.

A sensitivity analysis was undertaken to account for the fact that not all reference laboratory investigations had been completed (denominator = 220,985 AC2 tests). The nvCT/AC2neg prevalence estimates produced by the sensitivity analysis (0.003% for England, 0.004–0.109% by region) were similar to the original estimates.

## Discussion

Despite extensive investigation, there is no evidence of the presence of F-nvCT in England to date. However, investigations did identify two previously unpublished variants that had resulted in false-negative AC2 tests. Prevalence of nvCT/AC2neg was estimated to be very low; likely fewer than 0.003% nationally and less than 0.1% at a regional level. These estimates should be considered in the context of a 0.3% false-negative yield expected from normal AC2 test performance assuming an overall sensitivity of 94.5% [7] and an observed CT prevalence of 5.8%. As so few individuals were likely to have received a false-negative test result before June 2019, a patient recall was not indicated.

Two other countries, Finland and Sweden, have published data regarding investigations to identify F-nvCT in their populations, confirming its presence [1,8]. Ten cases (denominator unknown) with F-nvCT identified in Helsinki and Turku, Finland were reported in April 2019. Based on their RLU values, 0.4% of 9,472 tested specimens (6% of CT-positives) in Turku were suspected to contain the variant in late 2018 [1]. In Örebro, Sweden, F-nvCT was identified in two discrepant specimens sequenced (1.3% of CT-positives) [8]. Other variants were not reported, but the number of discrepant specimens sequenced was much fewer than in England.

This new variant CT event follows the earlier Swedish variant-CT (swCT) in 2006 [9], where a 377-bp deletion in the 7.5-kb chlamydial cryptic plasmid prevented detection by two widely used NAATs [10]. Strains lacking the entire 7.5-kb chlamydial cryptic plasmid have been reported, most recently from Australia [11,12]. These variant CT events, including the current event, demonstrate genetic diversity within NAAT target sites. The swCT spread throughout Sweden and its ability to escape detection was thought to confer a survival advantage, increasing the strain reproductive rate [13]. However, only one to two cases of swCT were found in Norway, Denmark [14], Ireland [14] and France [15]. Our estimates of very low nvCT/AC2neg prevalence and the small number of cases of the 2006 swCT detected outside of Sweden suggests that transmission also depends on host population contact dynamics and health-seeking behaviour, as well as health service factors.

Though the emergence of further variants able to evade detection by specific NAATs may occur, adverse impacts of such may be largely prevented by using CT-diagnostic tests with multiple molecular targets. Surveillance methods could also be developed. National sentinel surveillance for mutant strains could be introduced, similar to the technique used in the Netherlands to monitor the potential introduction of swCT [16]. This would involve subjecting a sub-set of CT clinical specimens to alternative NAAT tests with different molecular targets, or a variant-specific NAAT. Statistical process control (SPC) methods could also be used to compare the observed percentage of CT-positive samples with those expected from historic data to flag a deviation. However, detecting a small number of cases potentially confined to only one region using SPC methods may be difficult as it requires reliable, standardised specimen and data flows. Conducting rapid, prospective, ad hoc surveillance to a regional geographical granularity when a new variant is suspected, the methods we describe here, is also an alternative approach.

Provided the FSN is adhered to, current reflex testing arrangements should prevent nvCT/AC2neg variants that prospectively arise or are introduced into England from escaping detection. This reflex testing should continue until a revised version of the AC2 test is deployed. Even so, further novel variants escaping CT-NAAT detection may arise. F-nvCT [1] and the variant recently reported from Australia [11] were discovered because of the recognition of discrepant CT results from different NAATs. Continued vigilance is required from clinicians and laboratories to recognise false-negative tests in the presence of symptoms *compatible* with chlamydia infection.
