# Addendum for Euro Surveill. 2019;24(38)

**DOI:** 10.2807/1560-7917.ES.2019.24.49.1912051

**Published:** 2019-12-05

**Authors:** Eurosurveillance editorial team

**Affiliations:** 1 European Centre for Disease Prevention and Control (ECDC), Stockholm, Sweden

In the article ‘Prevalence of new variants of *Chlamydia trachomatis* escaping detection by the Aptima Combo 2 assay, England, June to August 2019’ by DJ Roberts et al., published on 19 September 2019, GenBank accession numbers were added on 4 December 2019.

